# Supplementary material for: Paving the Way for CCK2R-Targeted Peptide Receptor Radionuclide Therapy with [177Lu]Lu-DOTA-MGS5 in Patients with Small Cell Lung Cancer
Source: Pharmaceutics. 2026 Jan 22;18(1):138. doi: 10.3390/pharmaceutics18010138 (PMC12844804; doi:10.3390/pharmaceutics18010138)
Supplement: Supplementary file 1 [file pharmaceutics-18-00138-s001.zip › pharmaceutics-4089550-supplementary.pdf]

Supplementary Materials

Paving the Way for CCK2R-Targeted Peptide Receptor Radionuclide Therapy with [^{177}Lu]Lu-DOTA-MGS5 in Patients with Small Cell Lung Cancer

Taraneh Sadat Zavvar [1], Giulia Santo [1], Leonhard Gruber [2], Ariane Kronthaler [1], Judith Hagenbuchner [3], Ira Skvortsova [4,5], Inken Piro [6], Katja Steiger [6], Vladan Martinovic [7], Danijela Minasch [8], Judith Löffler-Ragg [7], Gianpaolo di Santo [1], Irene J. Virgolini [1] and Elisabeth von Guggenberg [1,*]

[1] Department of Nuclear Medicine, Medical University of Innsbruck, 6020 Innsbruck, Austria

[2] Department of Radiology, Medical University of Innsbruck, 6020 Innsbruck, Austria

[3] 3D Bioprinting Core Facility, Department of Child and Adolescence Health, Pediatrics I, Medical University of Innsbruck, 6020 Innsbruck, Austria

[4] EXTRO-Lab, Department of Therapeutic Radiology and Oncology, Medical University of Innsbruck, 6020 Innsbruck, Austria

[5] Tyrolean Cancer Research Institute (TKFI), 6020 Innsbruck, Austria

[6] Institute of Pathology, TUM School of Medicine and Health, Technical University of Munich, 81675 Munich, Germany

[7] Department of Pulmonology, Hospital Natters, 6161 Natters, Austria

[8] Department of Radiation Oncology, Medical University of Innsbruck, 6020 Innsbruck, Austria

* Correspondence: elisabeth.von-guggenberg@i-med.ac.at

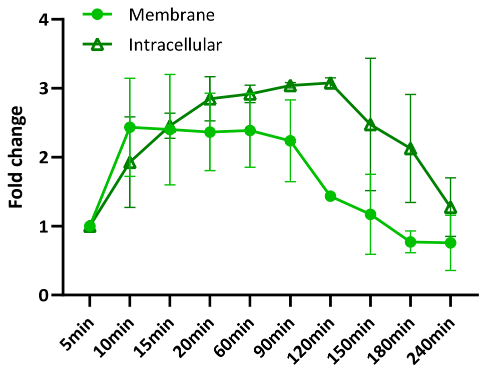

Supplementary Figure S1. Quantitative analysis of membrane-associated and intracellular fluorescence over time.

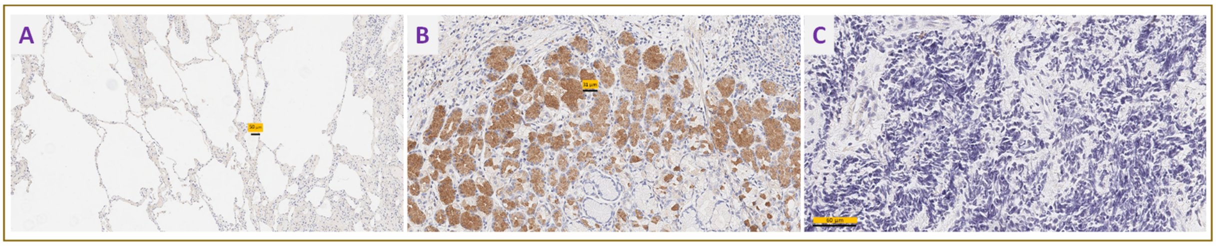

Supplementary Figure S2. CCK2R immunoreactivity: (A) normal lung tissue lacking CCK2R expression (magnification: ×100), (B) human stomach tissue known to physiologically express CCK2R (magnification: ×300), (C) anti-integrin β6 Mouse mAb staining of sample from patient 15 showing no tumor cell staining, indicating absence of nonspecific mouse IgG background (magnification: ×400).

Supplementary Table S1. Acceptance criteria and quality control results of four individual therapeutic batches of [^{177}Lu]Lu-DOTA-MGS5

Parameter	Method	Limits	Mean ± sd
Appearance	Visual inspection	Clear, colorless solution with no visible particulates	Conforms
pH	Indicator strip	5-7	6
Volume	Graduated vial (mL)	10-20	14.2 ± 0.2
Activity of the final product	MBq	>1000	6507.3 ± 193.4
Radioactivity concentration	MBq/mL	<700	458.2 ± 10.03
Radionuclide identity	Gamma-ray spectrometry (113 and 208 kev)	conforms	conforms
Identity of [^{177}Lu]Lu-DOTA-MGS5	HPLC (comparison with reference ^{nat}Lu-DOTA-MGS5)	0.9-1.1	conforms
Radiochemical purity	RCP (HPLC)	≥95%	97.9 ± 0.2
Free lutetium-177	TLC (0.1 sodium citrate pH 5) Rf 0.8-1.0	<1%	0.12 ± 0.08
Radiocolloid	TLC (1 M ammonium acetate/methanol; 1/1); Rf 0-0.3	<2%	0.09 ± 0.05
Limit test for peptide content	HPLC (UV)	≤100	69.14 ± 33.01
Apparent specific activity	MBq/µg	>20	65.07 ± 1.93
Ethanol content	Gas chromatography (v/v)	≤10%	6.05 ± 3.23
Bacterial endotoxins	LAL test (EU/V)	<175	<37
Sterility	Ph. Eur.	sterile	Sterile
Injected patient dose	MBq	-	4321.3 ± 213.2
Injected patient volume	Graduated vial	-	9.4 ± 0.6

Time-Activity Curve Fitting

Extrapolated time-activity curves were generated by fitting activity data obtained from quantitative SPECT imaging, normalized to the injected activity. A tri-exponential function was used for the kidneys, while a bi-exponential model (with $k_3 = 0$) was applied for the stomach wall and the two lesions.

Subsequently, the fitted curves were scaled to percent injected activity and normalized to organ or lesion mass (in grams), resulting in the following expression for the relative activity:

$$\boldsymbol{Activity}\left(\%\frac{\boldsymbol{IA}}{\boldsymbol{g}}\right) = \left(\boldsymbol{k_1} \times \boldsymbol{e^{-\lambda_1 t}} + \boldsymbol{k_2} \times \boldsymbol{e^{-\lambda_2 t}} + \boldsymbol{k_3} \times \boldsymbol{e^{-\lambda_3 t}}\right) \times \frac{\mathbf{100}}{\boldsymbol{mass(g)}}$$

Fitting was performed using nonlinear regression in Microsoft Excel, using the Solver add-in based on a least-squares approximation. This model was used to describe the kinetics of tracer clearance and to extrapolate time-activity data beyond the final measurement time point.

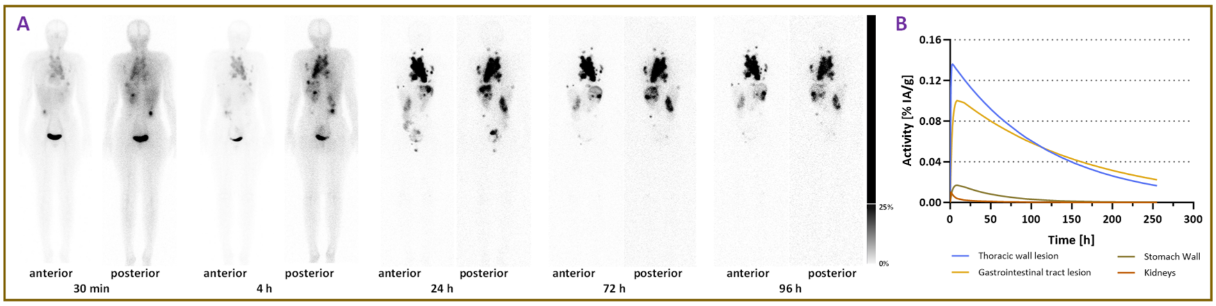

Supplementary Figure S3. Dosimetric evaluation with 1.5 GBq [^{177}Lu]Lu-DOTA-MGS5: (A) representative serial whole-body planar images of the patient with ED-SCLC, (B) time–activity curves extrapolated for two lesions, as well as for the stomach and kidney, were derived from the serial SPECT/CT scans.

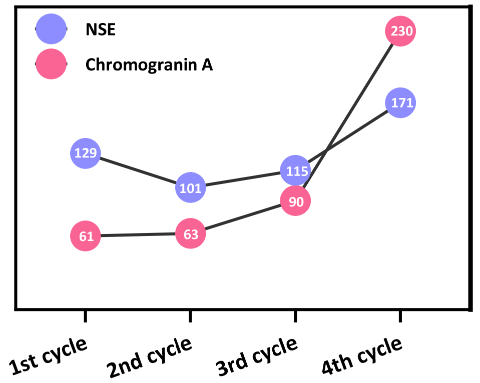

Supplementary Figure S4. Trend of tumor markers during PRRT with [^{177}Lu]Lu-DOTA-MGS5 (reported as ng/mL).

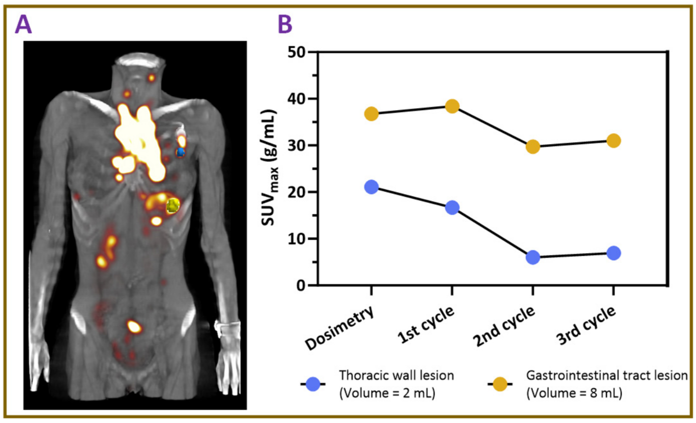

Supplementary Figure S5. (A) Fused coronal view of a whole-body SPECT/CT scan acquired 24 h p.i. of [^{177}Lu]Lu-DOTA-MGS5. The thoracic wall lesion is masked in blue, and the gastrointestinal tract lesion is masked in yellow. (B) A comparison of lesion uptake at 24 h p.i., indicated by SUV$_{max}$, is shown for both well-delineated lesions during dosimetry and the first three treatment cycles.

Image acquisition protocol after the last therapy cycle

Whole-body SPECT/CT after the last therapy cycle was performed using a dual-head SPECT/CT system (Symbia T, Siemens) 3.5 hours post-injection. The acquisition consisted of 64 projections over 360° with an angular step of 5.625° and a dwell time of 20 seconds per projection, using a medium-energy low-penetration (ME) collimator. Energy windows were set at 15% width around the photopeaks at 113

and 208 keV, each supplemented with upper and lower scatter windows for scatter correction. The SPECT images were reconstructed using the manufacturer's proprietary algorithm Flash 3D (OSEM with resolution recovery), applying 8 iterations and 4 subsets, and Gaussian filtering (FWHM = 9 mm). The reconstructed matrix size was 128 × 128 with a pixel size of 4.80 mm. Attenuation correction was performed using a low-dose CT scan acquired with a tube voltage of 130 kVp and an effective exposure of 17 mAs. Whole-body planar imaging was performed in anterior and posterior views using the same system. The scan was conducted in supine position, feet-first orientation. The table traversed 2 m at a speed of 15 cm/min. The matrix size was 1024 × 256 with a pixel spacing of 2.40 mm.

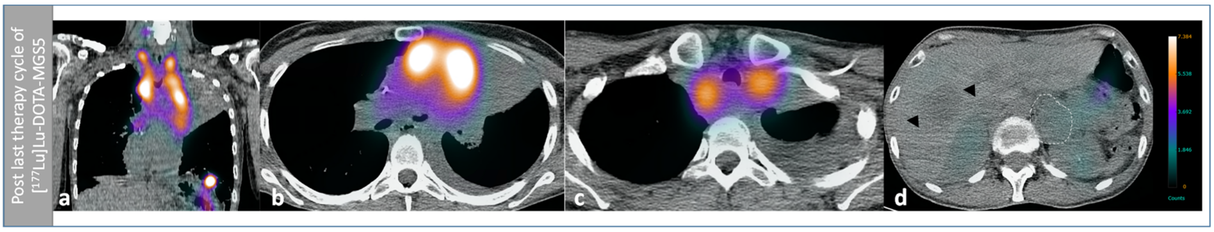

Supplementary Figure S6. Coronal and axial fused SPECT/CT images (a-c) after the fourth cycle of treatment with [^{177}Lu]Lu-DOTA-MGS5; next to stable uptake in CCK2R-positive disease new disseminated liver metastases without [^{177}Lu]Lu-DOTA-MGS5 uptake was noticed in the CT scan (black arrowheads; d).
